# High genetic diversity of HIV-1 *pol* region and molecular transmission networks among people living with HIV-1 in Haikou, South China, 2005–2022

**DOI:** 10.1186/s12879-025-11184-y

**Published:** 2025-07-01

**Authors:** Dee Yu, Mu Li, Liangjia Wei, Kaokao Zhu, Rongjing Zhang, Tong Luo, Yi Ning, Hao Liang, Jing Zhang, Li Ye, Bingyu Liang

**Affiliations:** 1 School of Public Health, Key Laboratory of Tropical Translational Medicine of Ministry of Education, Hainan Medical University, Haikou, Hainan 571199 China; 2 Guangxi Key Laboratory of AIDS Prevention and Treatment & Guangxi Colleges and Universities Key Laboratory of Prevention and Control of Highly Prevalent Diseases, School of Public Health, Guangxi Medical University, Nanning, Guangxi 530021 China; 3 The Fifth People’s Hospital of Hainan Province, Affiliated Dermatology Hospital of Hainan Medical University, Haikou, Hainan 570102 China; 4 Guangxi Engineering Center for Organoids and Organ-on-chips of Highly Pathogenic Microbial Infections & Biosafety III laboratory, Life Science Institute, Guangxi Medical University, Nanning, Guangxi 530021 China

**Keywords:** HIV-1, Subtype, Molecular epidemiology, Transmission network, Centrality analysis

## Abstract

**Background:**

Hainan is experiencing a continuous increase in newly diagnosed HIV-1 infections, highlighting the need for a comprehensive understanding of local transmission dynamics. This study aims to elucidate the genetic diversity and potential HIV-1 molecular transmission networks among people living with HIV-1 in Hainan, China.

**Methods:**

We used the HIV-TRACE to infer the transmission dynamics of HIV-1 at a 1.5% gene distance threshold. The role of HIV-1 diversity in transmission networks was assessed through node influence measurement and centrality analysis.

**Results:**

A total of 986 *pol* sequences were included, with CRF07_BC (43.71%) and CRF01_AE (37.12%) emerging as the predominant subtypes. Of these, 586 (59.43%) were clustered into the transmission networks, forming 83 clusters with 155 nodes having high transmission network scores (HTNS). CRF07_BC (adjusted odds ratio, *aOR*: 1.585, 95%*CI*: 1.189– 2.113) and CRF65_cpx (*aOR*: 9.513, *95%CI*: 3.694– 24.499) were more likely to cluster in networks and exhibit nodal centrality than CRF01_AE. The CRF65_cpx (88.46%) were more likely to be HTNS (*aOR*: 57.302, 95%CI:16.869– 194.643) than CRF07_BC.

**Conclusion:**

This study highlights the high genetic diversity of HIV-1 and its central role in transmission networks, advocating for targeted prevention strategies and community engagement for high-risk transmission populations, particularly focusing on subtypes CRF07_BC and CRF65_cpx.

**Supplementary Information:**

The online version contains supplementary material available at 10.1186/s12879-025-11184-y.

## Introduction

Human immunodeficiency virus type 1 (HIV-1) is subject to constant evolution, resulting in a continuous accumulation of genetic diversity across various regions globally. Furthermore, new variants continue to emerge, particularly in areas where multiple subtypes are circulating [1]. The high genetic diversity in HIV/AIDS contributes to varied responses to antiretroviral therapy (ART) [2]. In China, the distribution of HIV-1 subtypes is notably diverse and complex. The latest nationwide molecular epidemiological surveillance conducted in China in 2023 identified seventy-nine circulating recombinant forms (CRFs) of HIV-1, which accounted for 88.5% of the cases. Notably, these variants exhibit distinct geographical distributions across seven geographical regions of China from 2020 to 2023 [3].

Due to the ongoing evolution of HIV-1, individuals with genetically similar viral strains may be closely related by transmission. A molecular cluster is a group of individuals infected with similar HIV-1 strains. Identifying molecular clusters provides a novel approach to pinpoint transmission clusters. Integrating viral genetic, geospatial, and epidemiological data into dynamics models can offer insights into the migration history of pathogens and their drivers. This approach is particularly valuable when detailed contact tracing data is lacking and when connections between infections are not immediately evident [4]. Various approaches for prioritizing HIV-1 genetic clusters for intervention have been documented. These encompass the identification of rapidly growing clusters [5], clusters associated with new incident cases [6], and clusters characterized by drug resistance and specific sexual practices [7]. Insights into these molecular transmission clusters and the associated risk networks can assist us in directing proven HIV-1 prevention tools where they are most needed.

Hainan, the southernmost province of China, is noted for its tropical climate and attracts tourists from many countries. Surveillance data on the HIV/AIDS epidemic indicate that the annual number of newly reported HIV/AIDS cases in Hainan Province exhibited an overall increasing trend during 2004–2024, with fluctuating patterns observed in recent years. Haikou, the provincial capital, has the largest population, accounting for the largest case count in Hainan Province. The transmission patterns of HIV-1 in Haikou exhibit distinct characteristics. While heterosexual transmission plays an important role at the national and provincial levels, the epidemic in Haikou City is predominantly concentrated in men who have sex with men (MSM) [8]. Moreover, the MSM population demonstrates high mobility and often has multiple sexual partners. This behavior contributes to an elevated risk of generating HIV-1 CRFs and enhances the potential for cross-regional transmission, thereby presenting a challenge for HIV-1 prevention. Over the past decade, there has been a lack of comprehensive investigations on the HIV-1 subtypes and molecular transmission networks among HIV-1 patients in Haikou City and Hainan Province. To address these knowledge gaps, we conducted a cross-sectional study.

## Methods

### Study setting and participants

We conducted a cross-sectional study from January 2021 to December 2022 at the Fifth People’s Hospital of Hainan Province, the premier HIV/AIDS clinical treatment institution in this province. This hospital provides HIV-1 care to over 1500 people living with HIV (PLWH) in Haikou, representing more than 80% of PLWH in Haikou, and accounting for 59.23% in Hainan Province [8]. In this study, we used driven sampling approaches for participant enrollment. The inclusion criteria of participants were as follows: (1) age ≥ 18 years, (2) confirmed diagnosis of HIV-1 infection, and (3) signed informed consent. Approximately 10 mL of venous blood was taken from participants for laboratory testing. As of December 31, 2022, a total of 1704 individuals were documented as PLWH in Haikou City. We successfully recruited 1200 of participants, representing 70.42% (1200/1704) of the PLWH population. Finally, 986 *pol* sequences were successfully obtained (82.17%, 986/1200), representing a valid sample of the PLWH population in Haikou.

### Data sources

We obtained the participants’ demographic information at HIV-1 diagnosis, including sex, age, occupation, risk factor, education, marital status, ethnicity, HIV-1 diagnosis dates, baseline CD4 + T cell count, viral load, and ART initiation dates from the Infectious Disease Surveillance System of the China Information System for Disease Control and Prevention. Trained public health investigators conducted the investigation, followed up with patients using the national unified questionnaire.

### HIV-1 nucleic acid extracting and sequencing

We extracted nucleic acid from 200 µL of venous blood using the NP968 Nucleic Acid Extraction System (TIANLONG, Xi’an, China), at the GeneRotex 96 Nucleic Acid Extraction workstation of Guangxi Key Laboratory of AIDS Prevention and Treatment (Guangxi Medical University, Guangxi, China). And amplified the entire protease and a segment of the reverse transcriptase (nucleotides 2086 to 3441 in reference strain HXB2, 1356 bp in length) of HIV-1 *pol* gene using the Reverse Transcription-Polymerase Chain Reaction (RT-PCR) with combination primers for sequencing by Sangon Biotech Company [9, 10].

### Identification of HIV-1 subtypes and phylogenetic analysis

In this study, we assemble the *pol* sequences using Sequencher v5.4.6, and align them to an HXB2 sequence using an online tool available on the Los Alamos HIV Sequence Database (LANL, http://www.hiv.lanl.gov/). Then we extracted the *pol* segments (HXB2 positions 2253–3313) using BioEdit v7.2.5. The LANL online tool performed sequence quality control, after which we excluded sequences with ≥ 5% mixed bases or lengths ≤ 1000 bp.

For HIV-1 subtyping, we first classified sequences preliminarily using two online tools (COMET, https://comet.lih.lu, and REGA v3.46, https://www.genomedetective.com/app/typingtool/hiv) and then confirmed subtypes by constructing a maximum likelihood (ML) phylogenetic tree with LANL-downloaded near-full-length reference sequences. We built the ML tree under the general time reversible (GTR) model with gamma-distributed rate variation using IQ-TREE v1.6.12, selecting the best-fit model via the Akaike Information Criterion (AIC). We assigned subtypes to clusters with bootstrap values > 90% (0.90) matching reference sequences. Sequences unassignable to specific subtypes were designated unique recombinant forms (URFs). Finally, we visualized the ML tree using FigTree v1.4.4.

### Molecular transmission analysis

In this study, we selected the genetic distance threshold of 1.5% substitutions per site to construct molecular transmission networks using *pol* sequences, utilizing the HIV TRAnsmission Cluster Engine (HIV-TRACE). First, we aligned all HIV-1 *pol* sequences to the HXB2 reference (GenBank accession K03455) and calculated pairwise genetic distances under the TN93 nucleotide substitution model. We then inferred the transmission network from these pairwise distances.

Within the network, we keyed all nodes on either sequence names or their components extracted by regular expressions. We established links between individuals when any paired sequences showed genetic distances below the 1.5% threshold. We defined the number of links per node as its “degree” and considered any connected component with ≥ 2 sequences as a cluster. As previous studies have shown that codons of drug resistance does not affect clustering when using HIV-TRACE. We computed global network statistics, including node counts, links, clusters, cluster sizes, and degree distribution. All nodes in the network were ranked by their degrees, and nodes with degrees exceeding the 75th percentile (P_75th_) were defined as those with a high transmission network score (HTNS) [11]. The number of nodes in the clusters indicates the size of the cluster. Finally, we visualized the complete network using Cytoscape v3.10.0.

### Analysis of transmission sub-clusters

This method has been recently applied to construct local molecular transmission networks, which have been proven effective in providing insights into molecular transmission. We calculated the positions of the nodes within the transmission clusters using nodal centrality indicators (degree, closeness, betweenness, clustering coefficient, and radiality). We performed these computations with NetworkAnalyzer v2.7 [12], implemented in Cytoscape v3.10.0. Centrality refers to the degree to which a node is interconnected with other nodes within a network. Degree centrality quantifies the number of direct connections a particular node has, serving as a measure to estimate the average degree of connectivity within the network. This metric can be conceptualized as the extent of a node’s immediate influence. Closeness centrality is commonly employed to evaluate the speed at which a virus propagates from a particular node to other accessible nodes within a network. Betweenness centrality evaluates the frequency with which a node appears on the shortest paths between other nodes in the network [13]. Radiality, a measure of node centrality, is calculated by subtracting the average shortest path length of a given node from the sum of the diameter of the connected component and one [12]. The clustering coefficient quantifies the ratio of the actual number of connections among the neighbors of a given node to the maximum possible number of connections, and reflects the tightness of the local network of a node.

We identified the hub nodes using the K-shell (Ks) score [14]. Individuals with high Ks are considered to be the most influential in spreading and more susceptible to infection during epidemics. Molecular Complex Detection (MCODE), a novel graph theoretic clustering algorithm, was implemented into Cytoscape v3.10.0 to identify the densely connected sub-clusters within the large HIV-1 transmission networks [15]. The results of ranking and scoring indicate that larger, and more densely interconnected nodes occupy higher positions in the molecular network outcomes. We selected the sub-networks with the scores exceeding 5.0 for analysis.

### Data analysis

In this study, we categorized missing information using the multiple imputation method. We described continuous variables with medians and interquartile ranges (IQRs) and displayed categorical variables as numbers and proportions. We estimated associations between demographic variables and subtype categories using chi-square or Fisher’s exact tests. For sub-cluster centrality indicators in the network, we performed analyses with the Kruskal-Wallis test. To identify significant factors, we constructed multivariate logistic regression models. We considered variables with *P* < 0.05 statistically significant and reported them descriptively, including adjusted odds ratios (aORs) with 95% confidence intervals (95% CIs). Finally, we conducted all data analyses using SPSS v27.0.

## Results

### The socio-demographics of participants

Table 1 presents the demographic characteristics of 986 patients. Males accounted for 92.60% of participants, with a median diagnosis age of 29 years. Most patients were single (66.94%), had attained junior college or higher education (51.83%), and lacked permanent employment (56.19%). Regarding transmission routes, MSM accounted for 69.27% of cases, followed by heterosexual contact transmission (HETs, 22.31%). Baseline CD4 + T cell counts were between 201 and 499 cells/µL in 59.94% of patients. Half of the participants (50.60%) initiated ART within one month after diagnosis, while 23.43% developed drug resistance.

**Table 1:** Demographic and clinical characteristics of HIV-1 subtype from 2005 to 2022 in Haikou, China (N, %)

Variables	Total	CRF01_AE	CRF07_BC	CRF65_cpx	Others
**Total**	986(100.00)	366(37.12)	431(43.71)	57(5.78)	132(13.39)
**Sex**					
Female	73(7.40)	39(10.66)	19(4.41)	2(3.51)	13(9.85)
Male	913(92.60)	327(89.34)	412(95.59)	55(96.49)	119(90.15)
**Diagnosis age (year)**,** the median:29.0**,** inter-quartile ranges: 24.0– 39.0**					
≤ 29	513(52.03)	187(51.09)	233(54.06)	33(57.89)	60(45.45)
30– 39	234(23.73)	84(22.95)	104(24.13)	17(29.82)	29(21.97)
40– 49	134(13.59)	51(13.93)	61(14.15)	6(10.53)	16(12.12
≥ 50	105(10.65)	44(12.02)	33(7.66)	1(1.75)	27(20.45)
**Ethnicity**					
Han	908(92.09)	341(93.17)	387(89.79)	52(91.23)	128(96.97)
Others	78(7.91)	25(6.83)	44(10.21)	5(8.77)	4(3.03)
**Education level**					
Junior school and below	244(24.75)	101(27.60)	96(22.27)	7(12.28)	40(30.30)
High school	231(23.43)	81(22.13)	97(22.51)	18(31.58)	35(26.52)
University and above	511(51.83)	184(50.27)	238(55.22)	32(56.14)	57(43.18)
**Occupation**					
Regular worker	432(43.81)	171(46.72)	186(43.16)	15(26.32)	60(45.45)
Casual worker	554(56.19)	195(53.28)	245(56.84)	42(73.68)	72(54.55)
**Marital status**					
Divorced/widowed	77(7.81)	35(9.56)	26(6.03)	5(8.77)	11(8.33)
Single	660(66.94)	229(62.57)	313(72.62)	45(78.95)	73(55.30)
Married	249(25.25)	102(27.87)	92(21.35)	7(12.28)	48(36.36)
**Risk factors**					
Homosexuals	683(69.27)	229(62.57)	323(74.94)	50(87.72)	81(61.36)
Heterosexuals	220(22.31)	105(28.69)	77(17.87)	3(5.26)	35(26.52)
Others	83(8.42)	32(8.74)	31(7.19)	4(7.02)	16(12.12
**Diagnosis year**					
2005– 2014	245(24.85)	118(32.24)	95(22.04)	3(5.26)	29(21.97)
2015– 2018	381(38.64)	151(41.26)	156(36.19)	29(50.88)	45(34.09)
2019– 2022	360(36.51)	97(26.50)	180(41.76)	25(43.86)	58(43.94)
**Baseline CD4 + T counts (cells/µL)**,** the median:289.0**,** inter-quartile ranges:117.5– 389.0**					
1– 200	287(29.11)	136(37.16)	97(22.51)	15(26.32)	39(29.55)
201– 499	591(59.94)	198(54.10)	274(63.57)	38(66.67)	81(61.36)
≥ 500	108(10.95)	32(8.74)	60(13.92)	4(7.02)	12(9.09)
**Sampling CD4 + T counts (cells/µL)**,** the median:487.0**,** inter-quartile ranges: 329.5 - 646.5**					
1– 200	98(9.94)	55(15.03)	25(5.80)	5(8.77)	13(9.85)
201– 499	423(42.90)	148(40.44)	181(42.00)	26(45.61)	68(51.52
≥ 500	465(47.16)	163(44.54)	225(52.20)	26(45.61)	51(38.64)
**Sampling viral load**					
Lower than the detectable level	671(68.05)	248(67.76)	302(70.07)	32(56.14)	89(67.42)
Higher than the detectable level	187(18.97)	71(19.40)	73(16.94)	16(28.07)	27(20.45)
Undetectable	128(12.98)	47(12.84)	56(12.99)	9(15.79)	16(12.12)
**Duration from HlV-1 diagnosis to ART initiation**, **m**					
< 1	499(50.61)	159(43.44)	228(52.90)	39(68.42)	73(55.30)
≥ 1	487(49.39)	207(56.56)	203(47.10)	18(31.58)	59(44.70)
**ART or not**					
No	162(16.43)	47(12.84)	75(17.40)	12(21.05)	28(21.21)
Yes	824(83.57)	319(87.16)	356(82.60)	45(78.95)	104(78.79)
**Drug resistance**					
No	755(76.57)	276(75.41)	367(85.15)	5(8.77)	107(81.06)
Yes	231(23.43)	90(24.59)	64(14.85)	52(91.23)	25(18.94)

Note: *P *value was conducted using chi-square test or Fisher’s exact test

### High genetic diversity

**Fig. 1:**
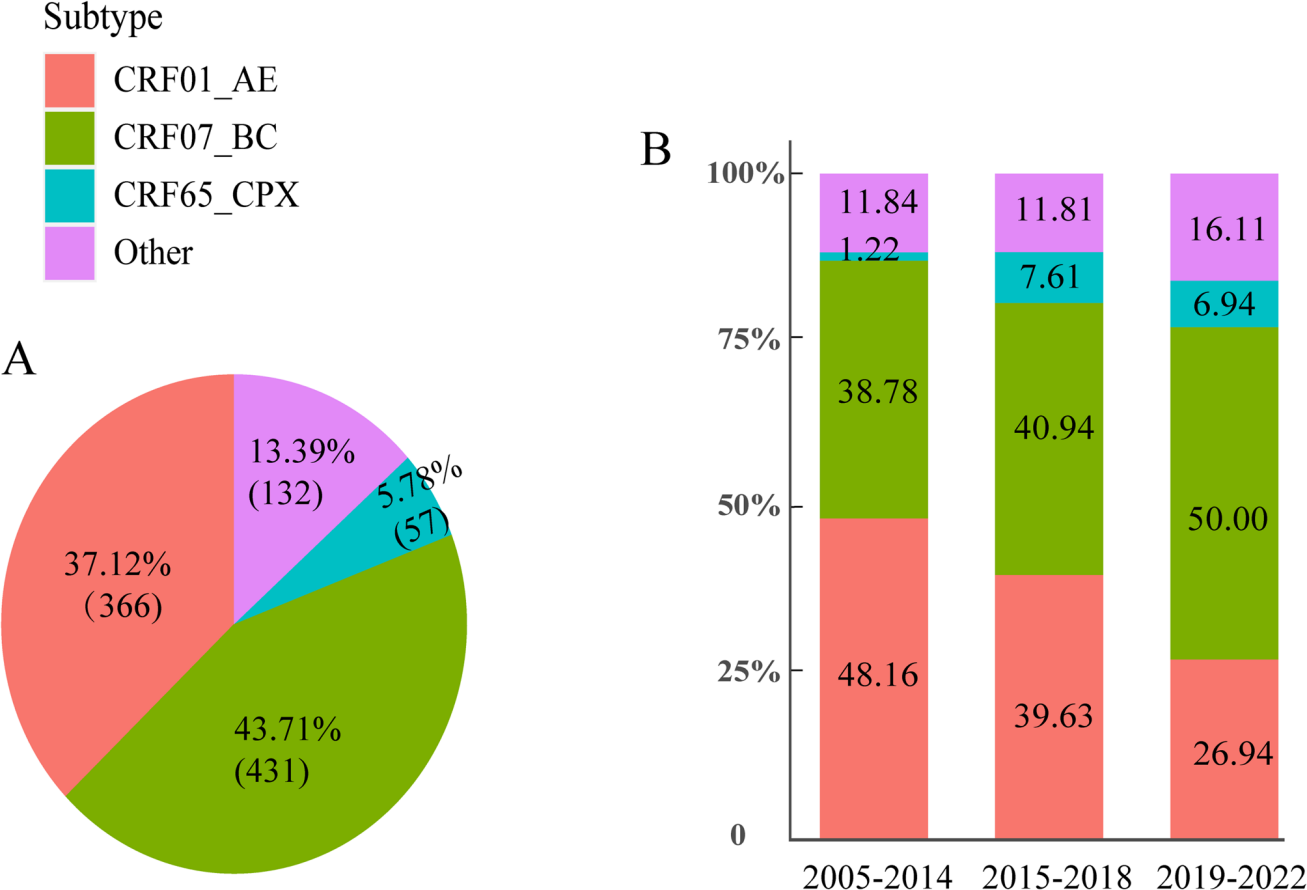
The proportions of HIV-1 subtypes in Haikou from 2005 to 2022. (**A**) The pie graph represents the proportions of HIV-1 subtypes. (**B**) The bars represent the percentage of HIV-1 subtypes over time. Others include subtype B, subtype C, CRF08_BC, CRF55_01B, CRF59_01B, CRF85_BC, CRF02_AG, CRF79_0107, CRF106_cpx, CRF102_0107 and URFs

Analysis of the ML tree (Supplementary Fig. 1) and Fig. 1 confirmed high genetic diversity of HIV-1 strains in Haikou. Among the 986 participants with available *pol* sequences, a total of 12 subtypes were identified, including 10 CRFs. CRF07_BC (43.71%) and CRF01_AE (37.12%) dominated the subtype distribution, followed by CRF65_cpx (5.78%). Between 2005 and 2022, CRF01_AE prevalence declined from 48.16 to 26.94%, while CRF07_BC increased from 38.78 to 50.00%. We detected CRF02_AG, CRF79_0107, CRF106_cpx, and CRF102_0107 in Haikou City for the first time.

### The subtype distribution

Table 1 presents the demographic and clinical characteristics of different HIV-1 subtypes in Haikou. Participants with the CRF01_AE subtype showed the following characteristics: 51.09% were younger than 29 years at HIV-1 diagnosis, 62.57% reported MSM transmission, and 41.26% received their diagnosis between 2015 and 2018. The CRF07_BC group comprised 54.06% under age 29, 74.94% MSM cases, and 41.76% diagnoses occurring during 2019– 2022. Among CRF65_cpx cases, 57.89% were under 29 years old, 87.72% involved MSM transmission, and 50.88% were diagnosed in 2015– 2018. Our analysis revealed baseline CD4 + T cell counts of 201– 499 cells/µL in 54.10% of CRF01_AE cases, compared to 40.44% at sampling. Corresponding figures for CRF07_BC reached 63.57% (baseline) and 42.00% (sampled), while CRF65_cpx showed 66.67% (baseline) and 45.61% (sampled).

### The characteristics of the HIV-1 molecular transmission network

**Fig. 2:**
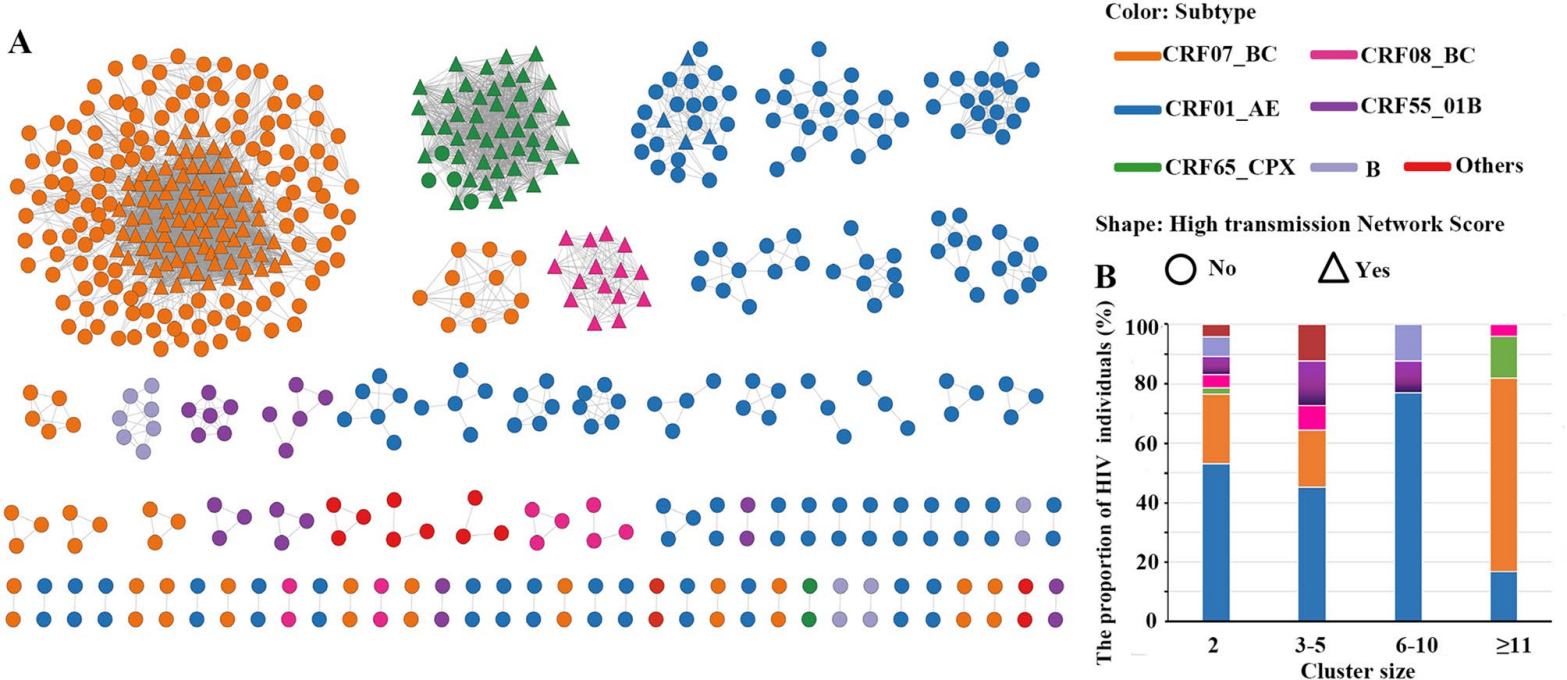
The HIV-1 molecular transmission network of patients during 2005– 2022 in Haikou, China. (**A**) The HIV-1 molecular transmission clusters were identified at a genetic distance threshold of 0.015 substitutions/site by HIV-TRACE. The shapes denote the nodes with high transmission network score (HTNS) in transmission network. (**B**) The x-axis denotes the size of HIV-1 transmission clusters. The y-axis shows subtype composition across differently sized clusters. Colors denote subtypes for both A and B

Among 986 *pol* sequences, 586 (59.42%) fell into the transmission networks and formed 83 clusters (the median size 2, range: 2– 225). One cluster contained more than 100 nodes (CRF07_BC), and 36 clusters (43.37%) contained ≥ 3 sequences. The proportions of cluster sizes of 3– 5, 6– 10, and ≥ 11 were 25.30% (21/83), 9.64% (8/83), and 8.44% (7/83), respectively. Out of the 83 clusters, the proportion of CRF01_AE, CRF07_BC, CRF65_cpx, CRF08_BC, CRF55_01B, and subtype B were 51.81%, 20.48%, 2.41%, 6.02%, 8.43%, and 4.82%, respectively (Fig. 2A). For the sequences located in the clusters comprising 2, 3– 5, and 6– 10 nodes, the majority were CRF01_AE, followed by CRF07_BC. Conversely, among the patients distributed in larger clusters (nodes ≥ 11), 65.19% were CRF07_BC, followed by CRF01_AE (19.75%) and CRF65_cpx (13.81%) (Fig. 2B).

### CRF07_BC and CRF65_cpx strains ongoing HIV-1 transmission in Haikou

**Fig. 3:**
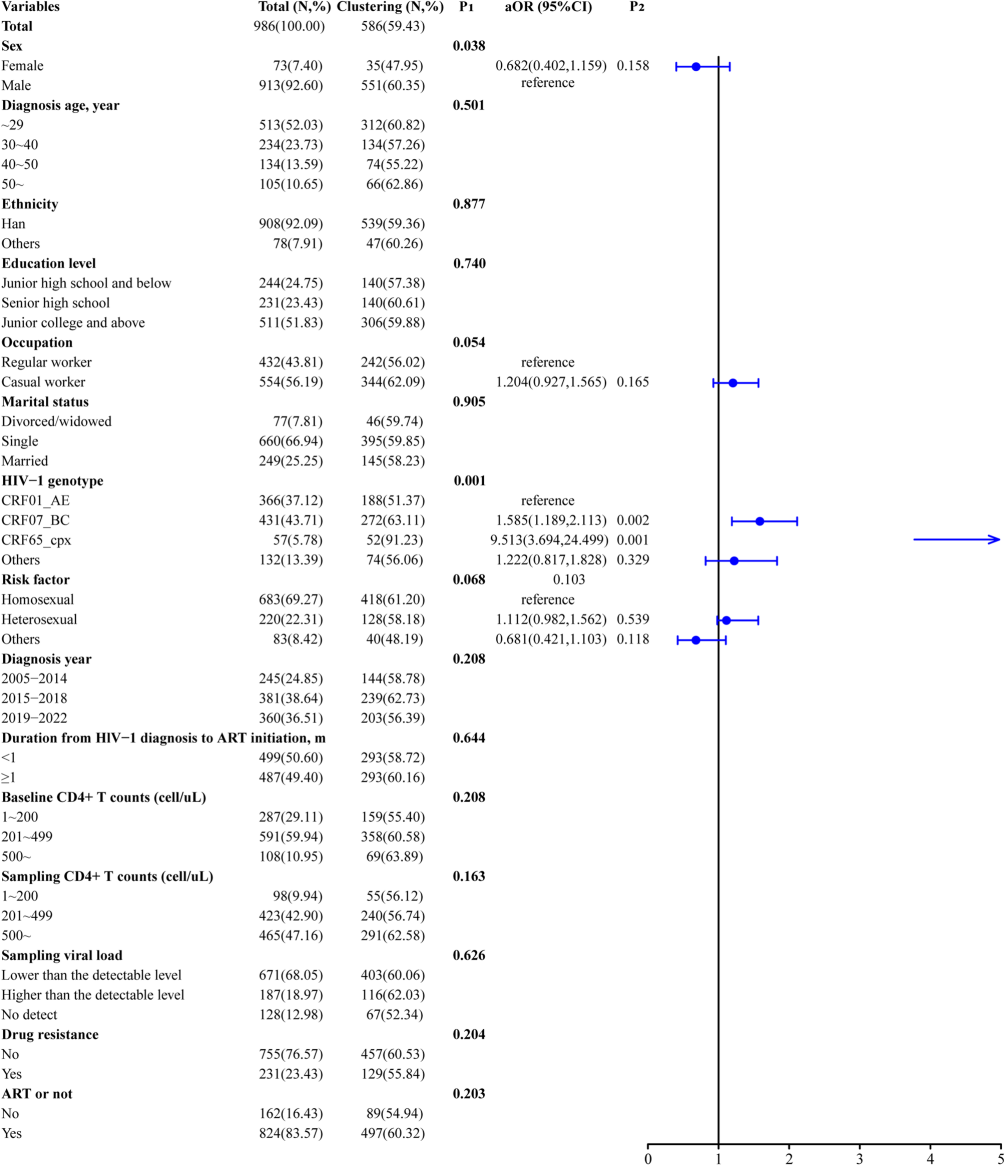
Clustering and non-clustering subjects in the molecular transmission network during 2005– 2022 in Haikou (*n* = 986). The “total” column indicates the number of participants in the Haikou surveillance population in that category, and the “clustering” column indicates the number and percentage of individuals in that category who were clustering. The P_1_ represents the significance of univariate analysis using chi-square test. The P_2_ indicates the significance of multivariable analysis using logistic regression model. The aOR means the results of multivariable analysis, including sex, occupation, HIV-1 genotype, and risk factor. ART, antiretroviral therapy. NRTI, nucleotide reverse transcriptase inhibitors. NNRTI, non-nucleoside reverse transcriptase inhibitor. PI, protease inhibitor. INSTI, integrase strand transfer inhibitor

Our analysis revealed that clustered and non-clustered sequences differed significantly (*P* < 0.05) by sex and subtype. Multivariable logistic regression analysis confirmed that subtype was the sole factor influencing sequences falling into the clusters. CRF07_BC (63.11%) and CRF65_cpx (91.23%) strains were more likely to cluster compared with CRF01_AE (51.37%) (*aOR*: 1.585, *95%CI*: 1.189– 2.113; *aOR*: 9.513, *95%CI*: 3.694– 24.499). Compared to females, males exhibited a higher risk of being clustered (60.35% vs. 47.95%); however, this difference was not statistically significant (*aOR*: 0.682, *95%CI*: 0.402– 1.159, *P* = 0.158) (Fig. 3).

### The determinants of patients with high transmission network score

**Fig. 4:**
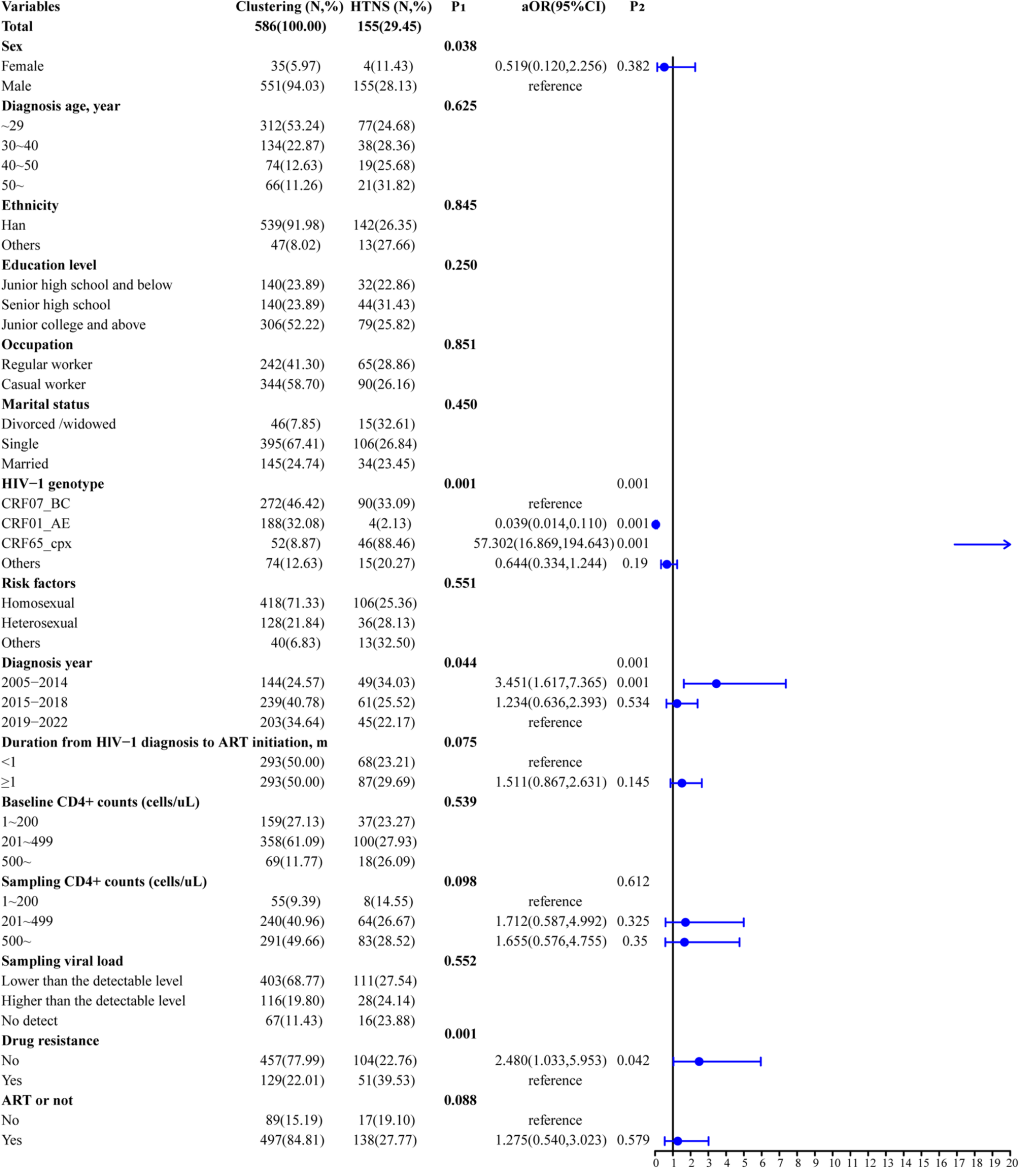
The factors associated with high transmission network score among HIV-1 patients in Haikou (*n* = 586). The “total” column indicates the number of populations in clusters in the category and the “HTNS” column indicates the number and percentage of individuals in the category who were HTNS. The P_1_ represents the significance of univariate analysis using chi-square test. The P_2_ indicates the significance of multivariable analysis using logistic regression model. The aOR means the results of multivariable analysis, including sex, HIV-1 genotype, diagnosis year, duration from HIV-1 diagnosis to ART initiation, sampling CD4^+^ counts (cells/µL), drug resistance, and ART or not. ART, antiretroviral therapy. NRTI, nucleotide reverse transcriptase inhibitors. NNRTI, nonnucleoside reverse transcriptase inhibitor. PI, protease inhibitor. INSTI, integrase strand transfer inhibitor

Figure 4 illustrates the characteristics of HTNS nodes. For the 586 sequences in the clusters, the median of node degree was one (range: 1– 115, P_25th_-P_75th_: 1– 14), and the number of the HTNS nodes was 155 (29.45%). Multivariable analysis showed that the patients diagnosed from 2005 to 2014 were more likely to be HTNS than those diagnosed from 2019 to 2022 (*aOR*: 3.451, *95%CI*: 1.617– 7.365). Notably, we observed that 88.46% (46/52) of CRF65_cpx in the clusters were HTNS. Compared with CRF07_BC, CRF65_cpx were more likely to be HTNS (*aOR*: 57.302, *95%CI*: 16.869– 194.643), and CRF01_AE were less likely to be HTNS (*aOR*: 0.039, *95%CI*: 0.014– 0.110).

### Centrality analysis of HIV-1 transmission networks for different subtypes

To further elucidate the molecular implications of HIV-1 subtype diversity in HIV-1 transmission, we performed centrality analysis. Our results showed that CRF07_BC and CRF65_cpx exhibited significantly higher centrality indicators compared with CRF01_AE, as evidenced by the elevated median degree (7.50 and 42.00 vs. 2.50), median radiality (0.99 and 0.99 vs. 0.95), and clustering coefficient (0.66 and 0.89 vs. 0.54) values (Table 2). These findings indicated a prominent role of CRF07_BC and CRF65_cpx strains in HIV-1 transmission networks.

**Table 2:** Centrality analysis of HIV-1 transmission networks for HIV-1 subtypes from 2005 to 2022 in Haikou, China

Centrality indicators	CRF01_AE	CRF07_BC	CRF65_CPX	Others	*P*
Number clusters (N, %)	43(51.81)	17(20.48)	2(2.41)	21(25.30)	
Closeness centrality	0.41(0.37, 0.46)	0.32(0.29, 0.35)	0.46(0.39, 0.54)	0.46(0.39, 0.54)	< 0.001
Clustering coefficient	0.54(0.48, 0.60)	0.66(0.61, 0.70)	0.89(0.84, 0.94)	0.55(0.45, 0.66)	0.006
Radiality	0.95(0.94, 0.96)	0.99(0.99, 0.99)	0.99(0.99, 1.00)	0.94(0.94, 0.98)	< 0.001
Betweenness centrality	0.028(0.016, 0.040)	0.005(0.003, 0.007)	0.004(0.002, 0.007)	0.028(0.005, 0.053)	< 0.001
K-shell scores of sub-networks	3.50(0.00, 5.00)	8.00(0.00, 50.852)	40.33(40.33, 40.33)	3.00(3.00, 15.00)	< 0.001
Degree	2.50(1.00, 5.00)	7.50(2.00, 27.75)	42.00(33.00, 45.00)	2.00(1.00, 5.00)	< 0.001
Number of seed nodes (N, %)	16(43.24)	12(32.43)	1(2.70)	8(21.62)	< 0.001

Note: *P* value was conducted using Wilcoxon signed-rank test

Notably, CRF07_BC (median Ks score: 8.00, IQR: 0.00– 50.85) and CRF65_cpx (40.33, IQR: 40.33– 40.33) showed statistically higher Ks scores than CRF01_AE (3.50, IQR: 0.00– 5.00) (Table 2). We then applied MCODE analysis to identify densely connected sub-clusters within larger transmission networks, detecting 37 key sub-clusters with 37 seed nodes. Of these, 13 sub-clusters had Ks scores ≥ 5.0 (containing 13 seed nodes). Among the 13 seed nodes, CRF07_BC accounted for 38.46% (Supplementary Fig. 2).

## Discussion

This study presents a pioneering effort in Hainan Province, revealing the characteristics of HIV-1 subtypes, and estimating the factors associated with HIV-1 transmission. The phylogenetic analyses strongly supported the high genetic diversity of HIV-1, with CRF07_BC, CRF01_AE, and CRF65_cpx being the most prevalent subtypes. The HIV transmission network supported by CRF07_BC and CRF65_cpx is experiencing rapid dissemination in Haikou. These findings provide valuable insights into the understanding of HIV-1 diversity in Hainan Province, with potential implications for enhancing local HIV prevention and control efforts.

Our study found that most HIV-1 patients in Haikou were MSM aged 20–29 years, similar to findings in Beijing [16] and Shenzhen [17], China’s most developed cities. As a prominent tourist destination, Hainan Province attracts a significant number of MSM population. A previous study found that the proportion of MSM among adult males in Hainan ranked fourth nationwide in China [18]. Within Hainan Province, the MSM community predominantly concentrated in economically thriving cities, with Haikou hosting a substantial MSM population. In these cities, there are community-based organizations (CBOs) that provide services to MSM, such as HIV-1 testing, counseling, and pre-exposure prophylaxis (PrEP) interventions. These factors may partly explain why HIV-1 cases in Haikou are mostly among MSM. These results highlight the need for targeted public health interventions in areas with varying transmission patterns.

As an international tourism destination, Hainan Province might have introduced multiple subtypes from different regions, which has increased the genetic diversity in this region. We identified CRF07_BC in 43.71% of isolates, with CRF01_AE being the second most prevalent subtype. CRF65_cpx showed comparable frequency to Beijing’s reports [19]. In the early stage of the HIV-1 epidemic in Haikou, the CRF01_AE was the most prevalent strain, particularly prevalent among HETs and intravenous drug users [20]. Currently, the CRF07_BC has emerged as the predominant strain among MSM populations. These findings were explained by three reasons: Firstly, the rise of CRF07_BC may be associated with slower HIV progression, which heightens the risk of transmission due to the asymptomatic phase and delayed diagnosis [21]; Secondly, the high mobility and multiple sexual partners within the MSM population may significantly contribute to the increased incidence of the CRF07_BC. Thirdly, HIV-related stigma might compel partially infected people to conceal their HIV-1 status [22], potentially resulting in unsafe sexual behaviors and further transmission of HIV. Therefore, it is imperative to strengthen surveillance of genetic diversity to understand transmission dynamics.

Interestingly, our network analysis revealed CRF07_BC had significantly stronger interconnectivity than CRF01_AE, aligning with a previous study conducted in Shenzhen [23]. CRF07_BC was also associated with the largest transmission clusters [24], and its distinct characteristics, including high immunogenicity [25], slower disease progression [26], and enhanced transmissibility [27], may explain why it is more prone to evolving into larger transmission clusters. Further analysis is needed to identify priority populations for intervention and understand the impact of different subtypes on clustering.

This study revealed a strong association between CRF65_cpx and HTNS, underscoring its pivotal role in HIV-1 transmission. CRF65_cpx, an emerging recombinant subtype described by Feng et al. [28], has become the predominant strain among the MSM population. Its expansion in Hainan may be attributed to the high prevalence of drug resistance [29], establishing it as the third most prevalent strain in Haikou City. Our previous research showed that MSM from Beijing introduced the CRF65_cpx strain to Hainan Island in 2013, leading to local transmission and making it the fourth most common subtype there [29]. In this study, each node represents an individual, with nodes exhibiting high HTNS indicating that the individual is at elevated transmission risk. Based on these findings, precise interventions should be implemented, encompassing enhanced treatment regimens, HIV-1 resistance testing, sexually transmitted infection screenings, and comprehensive education alongside behavioral interventions. For future research, it could be beneficial to incorporate innovative strategies, such as the utilization of digital tools for contact tracing and molecular surveillance expansion.

Our investigation into the prevalence patterns of HIV-1 in Haikou provides valuable insights; however, several limitations should be noted. Firstly, HIV-1 molecular clusters may not accurately reflect direct transmissions due to unsequenced or undiagnosed individuals. Secondly, relying on the *pol* sequence for genetic analysis might underestimate genetic diversity, suggesting the need for whole-genome sequencing in future research. Lastly, the cross-sectional nature of the study prevents assessment of HIV-1 transmission dynamics, highlighting the importance of longitudinal molecular surveillance for effective HIV-1 prevention and control in Hainan Province.

In summary, the study highlights the significant genetic diversity of HIV-1 in Haikou, with CRF07_BC and CRF65_cpx strains playing a key role in transmission. It indicates that individuals with these strains, diagnosed or not, continue to transmit the virus. Targeted interventions for these groups could be highly beneficial. The research underscores the importance of HIV molecular epidemiology in tourist areas with high population mobility, recommending future efforts in longitudinal studies, full-genome analysis, and drug resistance monitoring.

## Electronic supplementary material

Below is the link to the electronic supplementary material.

Supplementary Material 1

Supplementary Material 2

## Data Availability

The 986 HIV-1 sequences have been submitted to the HIV Sequence Database (http://www.hiv.lanl.gov/). The GenBank accessions of these sequences are: OP830908 - OP830910, OP830912, OP830916 - OP830918, OP830927, OP830936, OP830939 - OP830940, OP830942, OP830944, OP830948 - OP830949, OP830954 - OP830956, OP830966 - OP830968, OP830979, OP830985 - OP830987, OP830991 - OP830992, OP830994, OP831004 - OP831006, OP831010, OP831019 - OP831021, OP831023, OP831030, OP831034, OP831036 - OP831037, OP831039 - OP831040, OP831083 - OP831087, OP831100 - OP831103, OP831106, OP831115 - OP831120, OP831123 - OP831124, OP831132 - OP831134, OR606459 - OR606503, and PP270377 - PP271255. The public sequences used in the current study were downloaded from the HIV Sequence Database.

## Abbreviations

aOR: Adjusted odds ratio
ART: Antiretroviral therapy
CRFs: Circulating recombinant forms
MSM: Men who have sex with men
PLWH: People living with HIV
GTR: General time reversible
URFs: Unique recombinant forms
ML: Maximum likelihood
HIV-TRACE: HIV TRAnsmission cluster engine
HTNS: High transmission network score
MCODE: Molecular complex detection
CI: Confidence interval
CBOs: Community-based organizations
PrEP: Pre-exposure prophylaxis
HET: Heterosexual contact transmission
