# Supplementary material for: High genetic diversity of HIV-1 pol region and molecular transmission networks among people living with HIV-1 in Haikou, South China, 2005–2022
Source: BMC Infect Dis. 2025 Jul 1;25:813. doi: 10.1186/s12879-025-11184-y (PMC12210868; doi:10.1186/s12879-025-11184-y)
Supplement: Supplementary file 1 — Supplementary Material 1 [file 12879_2025_11184_MOESM1_ESM.docx]

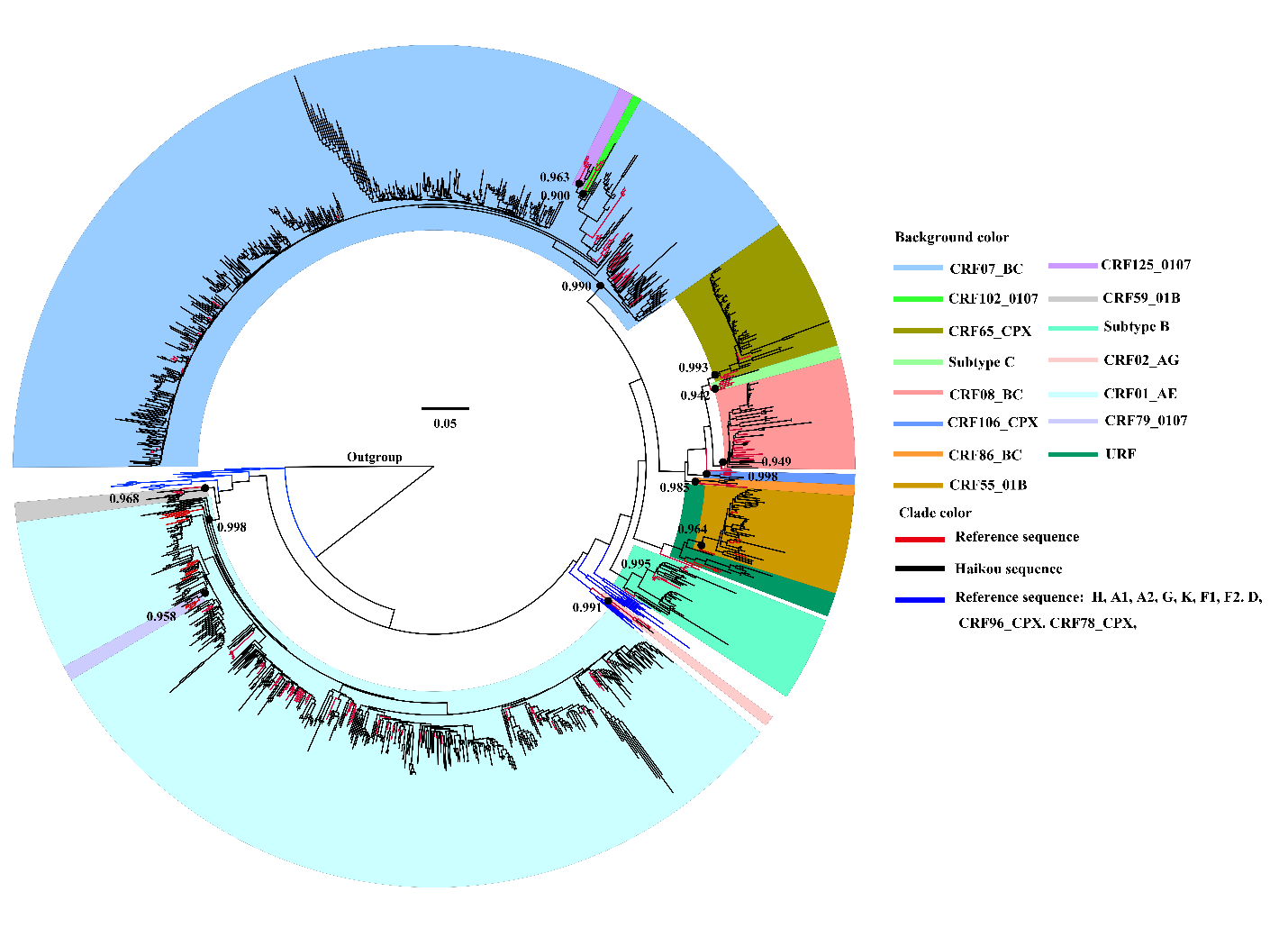


Supplementary Figure 1. The maximum likelihood phylogenetic tree (ML tree) of HIV-1 pol sequences obtained among HIV-1 patients during 2005–2022 in Haikou, China. A. The ML tree was constructed using 1197 HIV-1 pol sequences to identify subtype of Haikou HIV-1 sequences, including 986 Haikou sequences and 211 reference sequences. Background colors indicate subtypes. Clade colors represent sequence classification: reference sequences to be clustering with Haikou sequences (red), Haikou sequences (dark), and reference sequences of other subtypes (blue).
