# Supplementary material for: High genetic diversity of HIV-1 pol region and molecular transmission networks among people living with HIV-1 in Haikou, South China, 2005–2022
Source: BMC Infect Dis. 2025 Jul 1;25:813. doi: 10.1186/s12879-025-11184-y (PMC12210868; doi:10.1186/s12879-025-11184-y)
Supplement: Supplementary file 2 — Supplementary Material 2 [file 12879_2025_11184_MOESM2_ESM.docx]

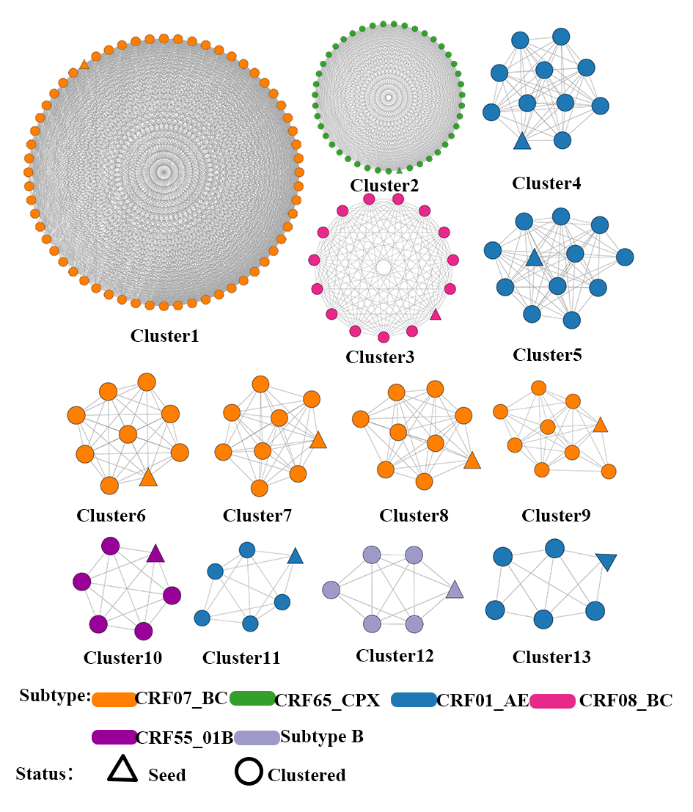


Supplementary Figure 2. The densely connected sub-clusters and seed nodes with Ks score ≥5.0 in large transmission clusters. Ks score was measured by a K-shell decomposition algorithm. The colors and shapes represent HIV-1 genotypes: CRF07_BC (yellow), CRF01_AE (blue), CRF65_cpx (green), CRF08_BC (pink), CRF55_01B (modena) and subtype B (lavender). The shapes indicator nodes status in clusters: clustered (circle), seed (triangle). Seed is the highest scoring node in the densely connected sub-clusters.
